# N-cadherin stabilises neural identity by dampening anti-neural signals

**DOI:** 10.1242/dev.183269

**Published:** 2019-11-01

**Authors:** Karolina Punovuori, Rosa P. Migueles, Mattias Malaguti, Guillaume Blin, Kenneth G. Macleod, Neil O. Carragher, Tim Pieters, Frans van Roy, Marc P. Stemmler, Sally Lowell

**Affiliations:** 1 MRC Centre for Regenerative Medicine, Institute for Stem Cell Research, School of Biological Sciences, The University of Edinburgh, Edinburgh EH16 4UU, UK; 2 Cancer Research UK Edinburgh Centre, MRC Institute of Genetics and Molecular Medicine, University of Edinburgh, Edinburgh EH4 2XR, UK; 3 Department of Biomedical Molecular Biology, Ghent University; Inflammation Research Center, VIB; Center for Medical Genetics, Ghent University Hospital; Cancer Research Institute Ghent (CRIG), Ghent B-9000, Belgium; 4 Department of Biomedical Molecular Biology, Ghent University; Inflammation Research Center, VIB; Cancer Research Institute Ghent (CRIG), Ghent B-9000, Belgium; 5 Department of Experimental Medicine I, Nikolaus-Fiebiger Center for Molecular Medicine, Friedrich-Alexander University of Erlangen-Nürnberg, Erlangen D-91054, Germany

**Keywords:** Cadherin, FGF, Neural development, Pluripotent, Wnt, Mouse

## Abstract

A switch from E- to N-cadherin regulates the transition from pluripotency to neural identity, but the mechanism by which cadherins regulate differentiation was previously unknown. Here, we show that the acquisition of N-cadherin stabilises neural identity by dampening anti-neural signals. We use quantitative image analysis to show that N-cadherin promotes neural differentiation independently of its effects on cell cohesiveness. We reveal that cadherin switching diminishes the level of nuclear β-catenin, and that N-cadherin also dampens FGF activity and consequently stabilises neural fate. Finally, we compare the timing of cadherin switching and differentiation *in vivo* and *in vitro*, and find that this process becomes dysregulated during *in vitro* differentiation. We propose that N-cadherin helps to propagate a stable neural identity throughout the emerging neuroepithelium, and that dysregulation of this process contributes to asynchronous differentiation in culture.

## Abstract (precis)

**Summary:** As pluripotent cells undergo neural differentiation they swap E-cadherin for N-cadherin. This switch in adhesion molecules modulates signalling in order to facilitate the differentiation process.

## INTRODUCTION

There is an increasing appreciation that changes in adhesion and morphology help to regulate cell fate changes (Gilmour et al., 2017). The homotypic adhesion molecule E-cadherin (also known as cadherin 1) is expressed on the surface of pluripotent cells and is downregulated and replaced with N-cadherin (cadherin 2) during early neural development (Hatta and Takeichi, 1986; Wheelock et al., 2008). We previously reported that loss of E-cadherin is not simply a consequence of differentiation, but rather that it actively promotes the neural differentiation process (Malaguti et al., 2013). However, the role of N-cadherin in this process and the mechanisms by which cadherins regulate neural differentiation are not known.

It has previously been reported that premature cadherin switching has profound effects at gastrulation, including an expansion of the extra-embryonic compartment, a reduction in the size of the epiblast, and mispatterning of the germ layers (Basilicata et al., 2016). These diverse phenotypes can be attributed, at least in part, to an overall reduction in BMP signalling within the epiblast and a reduction in pro-mesoderm signals at the primitive streak, which in turn may result from the gross morphological defects seen in these embryos (Basilicata et al., 2016). However, it is not clear which aspects of this complex phenotype are an indirect consequence of defects in extra-embryonic tissues and which, if any, are cell-autonomous. Here, we use cultured mouse pluripotent cells in order to focus on the mechanism by which cadherin switching influences neural differentiation of pluripotent cells in the absence of extra-embryonic tissues.

Mouse pluripotent cells can be cultured in the presence of inhibitors of MEK and Gsk3β plus LIF (2i-Lif) in order to maintain them in a naïve embryonic stem cell (ESC) state equivalent to the preimplantation epiblast (Boroviak et al., 2015; Ying et al., 2008), or they can be cultured in the presence of FGF and activin in order to maintain a differentiation-primed epiblast stem cell (EpiSC) state equivalent to the postimplantation epiblast (Brons et al., 2007; Nichols and Smith, 2009; Tesar et al., 2007). LIF and foetal calf serum (FCS) support a heterogeneous mixture of pluripotent cells moving in and out of the naïve state. We previously reported that cells downregulate E-cadherin during neural differentiation of ESCs, and that loss of E-cadherin leads to faster, more synchronous neural differentiation *in vitro* (Malaguti et al., 2013), in keeping with other reports that E-cadherin acts as a ‘brake’ to slow down differentiation of pluripotent cells (Chou et al., 2008; del Valle et al., 2013; Faunes et al., 2013; Livigni et al., 2013; Redmer et al., 2011; Soncin et al., 2009). E-cadherin-null ESCs display a loss of cell-cell adhesion (Larue et al., 1994, 1996), raising the possibility that their neural differentiation phenotype may be a secondary consequence of their adhesion defect. Alternatively, cadherins could influence differentiation by modulating signalling independently of adhesion (Bedzhov et al., 2012; del Valle et al., 2013; Wheelock et al., 2008; Zhang et al., 2010).

Neural specification depends on inhibition of BMP and Nodal signalling (Camus et al., 2006; Di-Gregorio et al., 2007). The ability of BMP to block neural fate is at least in part due to maintenance of E-cadherin expression, but it is not known which signalling pathways act downstream of cadherins to modulate differentiation. Dampening of either FGF (Greber et al., 2010; Jaeger et al., 2011; Stavridis et al., 2010; Sterneckert et al., 2010) or Wnt (Aubert et al., 2002; Haegele et al., 2003) has the effect of stabilising neural identity. N-cadherin has been reported to modulate FGF activity (Takehara et al., 2015; Utton et al., 2001; Williams et al., 1994, 2001) and E-cadherin has been reported to modulate Wnt activity in other contexts (Howard et al., 2011), and so it seems plausible that cadherin switching may modulate neural differentiation via dampening of one or both of these anti-neural signalling pathways. Alternatively, it is possible that cadherins modulate other signalling pathways (Pieters and van Roy, 2014).

Here, we set out to determine how the switch from E-cadherin to N-cadherin influences differentiation. We present evidence that N-cadherin promotes neural differentiation by dampening FGF activity. We also discover that cadherin switching occurs later and more synchronously during anterior neural differentiation *in vivo* compared with neural differentiation in culture. We suggest that cadherins could mediate a ‘community effect’ by helping to propagate differentiation decisions to neighbouring cells, and that this may help to ensure synchronous neural commitment in the embryo. This effect partly breaks down in culture, helping to explain why differentiation in culture is relatively asynchronous even in the face of a uniform extrinsic environment.

## RESULTS

### Cadherin switching is initiated prior to the onset of neural differentiation *in vitro*

We previously reported that E-cadherin inhibits neural differentiation, but the mechanism of action was not known (Malaguti et al., 2013). Upregulation of N-cadherin accompanies the loss of E-cadherin as pluripotent cells adopt a neural fate (Dady et al., 2012; Hatta and Takeichi, 1986), raising the possibility that N-cadherin might contribute to the regulation of the differentiation process. We first asked when N-cadherin becomes detectable during neural differentiation (note that we use the phrase ‘neural differentiation’ to mean the transition from pluripotency to neural identity rather than terminal differentiation into a particular neural derivative).

**Fig. 1.**
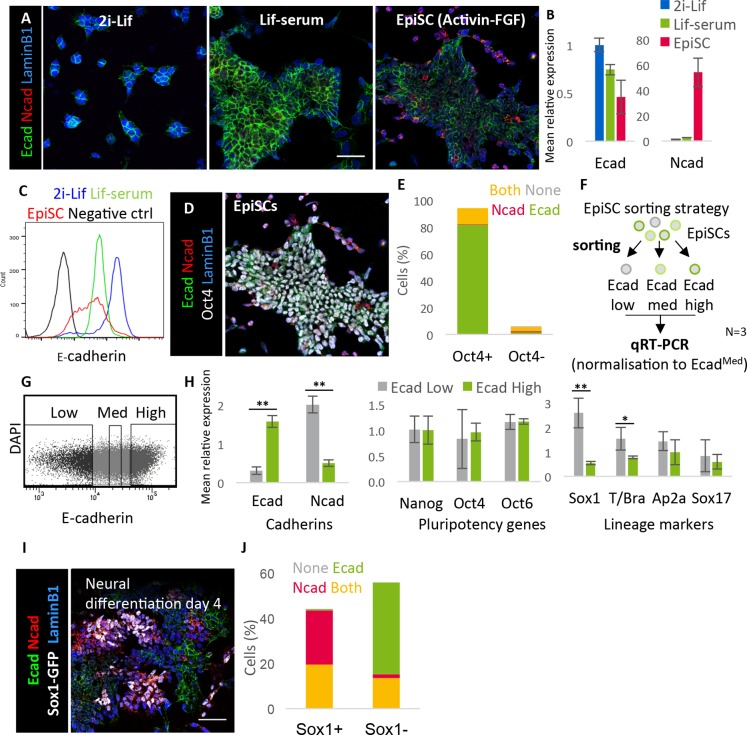
**Cadherin switching precedes the loss of pluripotency marker expression and coincides with neural priming *in vitro.*** (A) Cells cultured in three pluripotent conditions stained for E-cadherin, N-cadherin and the nuclear envelope marker lamin B1. (B) qRT-PCR analysis of E-cadherin and N-cadherin expression in cells cultured in three pluripotent conditions, *n*=3. (C) Flow cytometric analysis of E- and N-cadherin expression in cells cultured in various pluripotent conditions; curves show a representative sample of three biological replicates. (D) Example ICC image of EpiSCs stained for Ecad, Ncad, Oct4 and lamin B1. (E) Quantification of protein co-expression in EpiSCs. *n*=2596 cells from three biological replicates. (F) Sorting and analysis strategy for EpiSCs heterogeneously expressing E-cadherin. (G) Example FACS gating of EpiSCs into three populations based on their level of E-cadherin expression; each population makes up ∼20% of live cells. (H) qPCR analysis of sorted EpiSC populations. Ecad^Low^ and Ecad^High^ populations were normalised to the Ecad^Med^ population. *n*=3 independent sorts. (I) Example ICC image of Sox1-GFP (46C) cells undergoing neural differentiation stained for Ecad, Ncad, Sox1 and lamin B1. (J) Quantification of protein co-expression in Sox1-GFP cells undergoing neural differentiation. *n*=2275 cells from three biological replicates. Error bars represent s.d., *P≤0.05, **P≤0.01, paired Student's *t*-test. All images shown to same scale. Scale bars: 50 μm.

We confirmed that mouse ESCs cultured in 2i-Lif or Lif-serum express high levels of E-cadherin, whereas N-cadherin mRNA and protein were undetectable in either of these culture conditions (Fig. 1A,B). In EpiSC culture, E-cadherin expression was heterogeneous whereas N-cadherin became detectable in a subpopulation of cells (Fig. 1A-C). Cultures of EpiSCs contain spontaneously differentiating cells, and so we focused only on undifferentiated (Oct4^+^; also known as Pou5f1) cells (Fig. 1D, Fig. S1A). Almost all (99.6%) Oct4^+^ cells expressed E-cadherin and, of these, 13.0% also expressed N-cadherin (Fig. 1E). Very few (<1%) Oct4^+^ cells expressed N-cadherin alone. These results (Fig. S1) show that N-cadherin becomes expressed in a subpopulation of E-cadherin^+^ cells prior to loss of Oct4 expression.

Fluorescence-activated cell sorting (FACS) analysis confirmed that the vast majority of EpiSCs express E-cadherin, but revealed considerable cell-to-cell variability in the levels of this adhesion molecule on the cell surface (Fig. 1C, red curve). This contrasted with naïve pluripotent cells, which displayed uniformly high levels of E-cadherin throughout the population (Fig. 1C, blue curve). To further study cadherin heterogeneity in differentiation-primed cells, EpiSCs were sorted into three subpopulations (Ecad^High^, Ecad^Med^, and Ecad^Low^) (Fig. 1F,G). Ecad^High^ and Ecad^Low^ populations were then analysed by qRT-PCR, normalising to the Ecad^Med^ population (Fig. 1H). This analysis revealed a reciprocal expression pattern between E-cadherin and N-cadherin, consistent with an ongoing process of cadherin switching (Fig. 1H). The subpopulations expressed similar levels of the general pluripotency factors Oct4 and Nanog, and the primed pluripotency factor Oct6 (Pou3f1), indicating no difference in their overt differentiation status (Fig. 1H). It has been reported that differentiation-primed subpopulations of undifferentiated EpiSCs express low levels of either the neural-priming factor Sox1 or the mesoderm-priming factor T (brachyury) (Tsakiridis et al., 2014). We found that the E-cad^Low^ population expressed significantly higher levels of Sox1 and T, characteristic of a differentiation-primed subpopulation, whereas markers of surface ectoderm (Ap2a; Tfap2a) or endoderm (Sox17) did not differ significantly between the populations (Fig. 1H).

These results indicate that N-cadherin starts to become detectable prior to the loss of pluripotency transcription factors, and that a subpopulation of EpiSCs with lower E-cadherin and higher N-cadherin may be primed for neural and mesodermal differentiation.

We next examined cultures at an early stage of neural differentiation [day (D) 4], when around 50% of cells had started to adopt a neural identity, as revealed by immunostaining for Sox1-GFP (Ying et al., 2003), a reporter for the earliest marker of neuroepithelial identity (Wood and Episkopou, 1999) (Fig. 1I, Fig. S1B). In these cultures, N-cadherin was detectable in almost all Sox1-GFP^+^ neural cells, and of these around half also retained E-cadherin expression. Of cells that had not yet acquired a Sox1-GFP^+^ neural identity, almost all expressed E-cadherin, and of these around 20% also expressed N-cadherin (Fig. 1J).

Taken together, these results suggest that cadherin switching is initiated prior to the onset of neural differentiation *in vitro.*

### N-cadherin promotes neural differentiation

Loss of E-cadherin is associated with neural differentiation of ESCs but the mechanisms by which cadherins might influence neural differentiation are not known (Malaguti et al., 2013). One possibility is that this pro-neural differentiation phenotype of cells lacking E-cadherin function may be a secondary consequence of their adhesion defect (Larue et al., 1994, 1996). If this is the case, then restoring adhesion should restore normal neural differentiation capacity. N-cadherin can rescue the E-cadherin adhesion phenotype, at least in the context of preimplantation development (Bedzhov et al., 2012; Kan et al., 2007). We therefore asked whether providing N-cadherin to E-cadherin-null cells would rescue their neural differentiation phenotype.

**Fig. 2.**
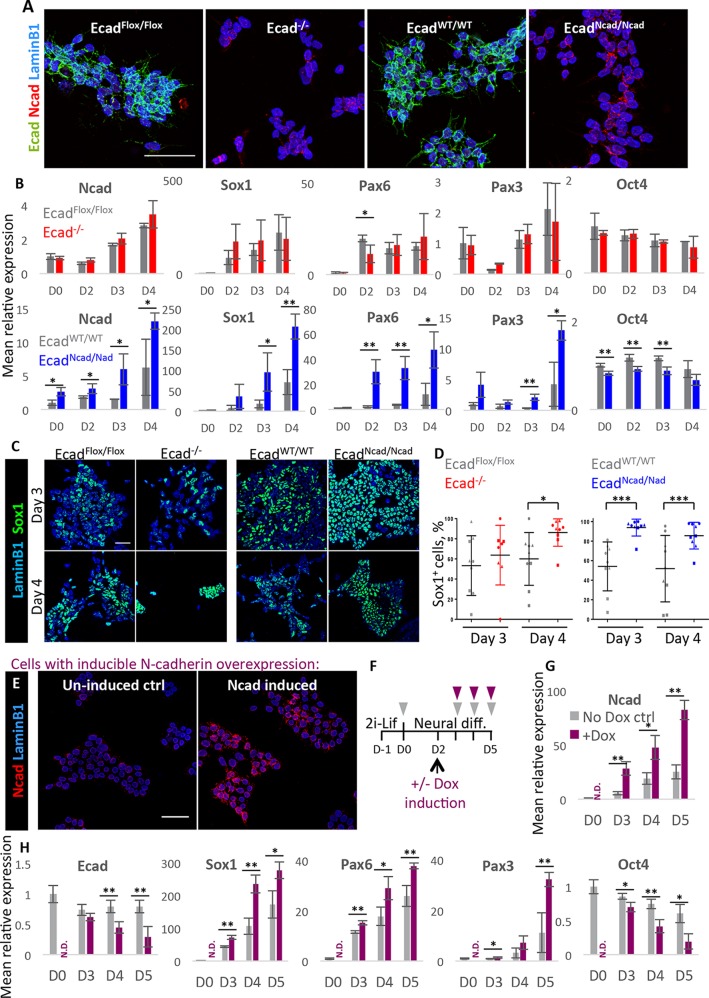
**N-cadherin promotes neural differentiation.** (A) Cadherin expression in four cell lines used for cadherin domain deletion/substitution experiments. Cells cultured in neural differentiation conditions for 24 h. Lamin B1: nuclear envelope marker. (B) qPCR analysis of the above cell lines during successive days of neural differentiation. *n*=3. Values normalised to control cell line on day (D)0. (C) Representative images showing expression of the early neural marker Sox1 in above cells after 3-4 days of neural differentiation. (D) Quantification of Sox1 expression in the above cells during neural differentiation. *n*=9, three fields of view from three biological replicates. (E) ICC of inducible N-cadherin-overexpressing cells cultured in neural differentiation conditions for 24 h with or without dox. (F) Protocol for neural differentiation of inducible N-cadherin-overexpressing cells. Triangles indicate sample collection. (G,H) qPCR analysis of inducible N-cadherin-overexpressing cells during neural differentiation; dox added on day 2. *n*=9 (three biological replicates of three independent clones). Error bars represent s.d., *P≤0.05, **P≤0.01, ***P≤0.001, unpaired Student's *t*-test. N.D., not determined. Scale bars: 50 µm.

To address this question, we made use of two E-cadherin knockout ESC lines: Ecad^−/−^, in which both cadherin alleles have been knocked out (Pieters et al., 2016), and an N-cadherin knock-in line, Ecad^Ncad/Ncad^, in which the coding sequence of N-cadherin is knocked into the E-cadherin locus, placing exogenous N-cadherin under the control of the endogenous E-cadherin regulatory elements and eliminating E-cadherin expression (Basilicata et al., 2016; Kan et al., 2007; Libusova et al., 2010) (Fig. 2A). The Ecad^Ncad/Ncad^ cells have been previously shown to enforce a cadherin switch while maintaining cell-cell adhesion in some developmental contexts (Kan et al., 2007). Because the two lines differ in their genetic background, they are analysed side-by-side with their relevant control cell lines. For the E-cadherin cell line, the control is the parental cell line in which the E-cadherin locus contains the loxP sites but recombination has not yet taken place (Ecad^Flox/Flox)^. For the E-cadherin^Ncad/Ncad^ cells, the wild-type (WT) control is the parental cell line in which the E-cadherin coding sequence is still intact (Ecad^WT/WT^).

We confirmed that N-cadherin was expressed above control levels in Ecad^Ncad/Ncad^ cells throughout the course of differentiation, but was not significantly elevated above background levels in Ecad^−/−^ cells (Fig. 2B).

Both Ecad^−/−^ and Ecad^Ncad/Ncad^ cells can be maintained in 2i-Lif conditions. When challenged with neural differentiation conditions, both cell lines were able to switch on the neural marker genes Sox1, Pax6 and Pax3 (Fig. 2B-D) (Pax3 is also expressed in the paraxial mesoderm, but is unlikely to be marking mesoderm in this context because the pan-mesodermal marker T is not upregulated above background levels in this set of experiments; Fig. S2). Ecad^−/−^ cells displayed a moderate pro-neural phenotype with some variability in neural gene expression (Fig. 2B-D). However, they showed greater instability than control cells, with very few cells surviving past d4 of neural differentiation (Fig. S3). In contrast, Ecad^Ncad/Ncad^ cells exhibited a more pronounced pro-neural phenotype, differentiating more rapidly than control cells (Fig. 2B-D). Quantification of Sox1 expression in individual cells indicated that although Sox1 shows considerable cell-cell variability in both control and (to a lesser extent) Ecad^−/−^ populations, this variability was largely eliminated in Ecad^Ncad/Ncad^ cells, which exhibited uniformly high Sox1 expression by D3 (Fig. 2C,D). These results indicated that exogenous N-cadherin reinforces rather than reverses the pro-neural phenotype of E-cadherin-null cells.

In order to test further whether N-cadherin contributes to promoting neural differentiation, we designed cell lines that allowed us to force expression of N-cadherin in the presence of endogenous E-cadherin. These cells, termed A2Lox-Ncad-HA cells, are engineered to enable doxycycline (dox)-inducible expression of an N-cadherin transgene with a C-terminal HA tag (Fig. 2E).

When exogenous N-cadherin was induced in these cells during neural differentiation (Fig. 2G), an increase in expression of the early neural markers Sox1, Pax6 and Pax3, and an accelerated downregulation of the pluripotency marker Oct4 was observed (Fig. 2H). E-cadherin was also downregulated more rapidly in the presence of ectopic N-cadherin, but these differences in E-cadherin did not emerge until 1 day after the pro-neural phenotype first became apparent. This might suggest that the loss of E-cadherin is likely to be a consequence rather than a cause of premature neural differentiation in these experiments, although we cannot exclude the possibility that changes in E-cadherin contribute to the effects on differentiation in these experiments.

Taken together, these results showed that the switch from E-cadherin to N-cadherin can promote neural differentiation under permissive conditions, and that the presence of N-cadherin contributes to this pro-neural effect.

### Pro-neural effects of N-cadherin are not explained by changes in cell cohesiveness

As the primary function of cadherins is in cell-cell adhesion, we assessed whether the pro-neural effects of cadherin switching might be a consequence of cells becoming less cohesive, i.e. moving further apart from one another. In culture, Ecad^−/−^ cells are unable to form large, compact colonies, instead growing as small dispersed clumps of a few cells (Fig. 2A) (Larue et al., 1994, 1996). By contrast, Ecad^Ncad/Ncad^ cells appear more similar to control cells (Fig. 2A) in keeping with the ability of N-cadherin to rescue the adhesion phenotype of E-cadherin-null blastocysts (Bedzhov et al., 2012; Kan et al., 2007). However, this does not exclude the possibility that manipulation of N-cadherin expression had subtle effects on cell cohesion that were not discernible by eye nor did it exclude the possibility that adhesion or migration defects became apparent under differentiation conditions.

**Fig. 3.**
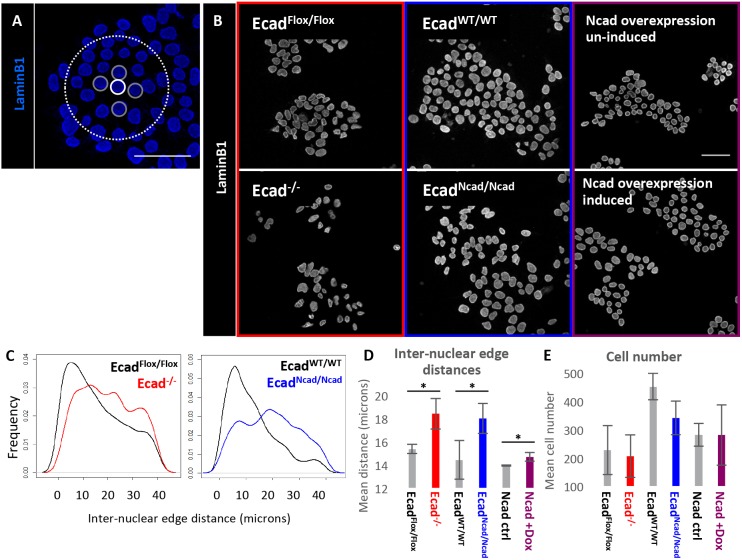
**Effects of cadherin switching on cellular clustering.** (A) Methodology for measuring inter-nuclear edge distances. For each nucleus (white solid line), the nearest neighbours (grey solid line) within a 40 µm radius (white dotted line) are determined by Delaunay triangulation, and the inter-nuclear edge distances between these nuclei are calculated; the process is repeated for all nuclei in the image. (B) ICC of all cell lines used for cadherin domain deletion/substitution experiments stained for lamin B1. Ncad overexpression was performed in A2Lox-Ncad-HA cells. Cells cultured in neural differentiation conditions for 24 h. (C) Density plots of inter-nuclear edge distance in four cell lines. *n*=1054 for all samples; plots show a representative sample for three biological replicates. (D) Mean inter-nuclear edge distances. *n*=3 biological replicates, each containing hundreds to thousands of cells. (E) Mean cell number. *n*=3 biological replicates. Error bars represent s.d., *P≤0.05, paired Student's *t*-test. Scale bars: 50 μm.

We set out to measure whether our manipulations of cadherin expression resulted in changes in cell cohesiveness. We measured the inter-nuclear distances of cells (Fig. 3A) because we are able to perform nuclear segmentation with high accuracy (Blin et al., 2019), and because pluripotent and early neural cells have very scant cytoplasm, so inter-nuclear distance is a reasonable proxy for inter-cellular distance. This assay was designed to capture indirectly the consequences of any changes in adhesion or morphology, which could include a relaxation of cell-cell contacts, an increase in cell spreading or cell volume, or an increase in migration. These measurements were performed in cells that had been cultured in neural differentiation conditions for 24 h; at this time, cells are starting to initiate differentiation but have not yet committed to a neural fate (Kalkan and Smith, 2014).

We demonstrated that Ecad^−/−^ cells had significantly greater mean inter-nuclear edge distances than control cells at 24 h under differentiation conditions (Fig. 3B-D), consistent with their previously reported adhesion defect (Larue et al., 1994, 1996). Surprisingly, Ecad^Ncad/Ncad^ cells also had higher mean inter-nuclear edge distances from one another compared with control cells under these conditions (Fig. 3C,D). The number of cells present in each analysis did not differ significantly between the control and experimental conditions, suggesting that the observed differences in clustering were not a result of variable cell density (Fig. 3E). These results indicate that, although N-cadherin can rescue the adhesion defect of Ecad^−/−^ cells in the context of preimplantation development (Kan et al., 2007), it cannot fully rescue effects on intercellular distances (which may result from changes in adhesion, migration and/or morphology) at early stages of neural differentiation in culture. These observations therefore do not exclude the possibility that the loss of E-cadherin influences differentiation partly though changes in adhesion, migration or morphology.

Exogenous N-cadherin can promote neural differentiation even in the presence of E-cadherin (Fig. 2G), and so we next asked whether these pro-neural effects of N-cadherin correlate with a change in inter-nuclear distances. When we induced N-cadherin in the presence of endogenous E-cadherin, inter-nuclear distances were barely affected (Fig. 3D). This alerted us to the possibility that the pro-neural effect of N-cadherin might not be fully explained by changes in cell cohesiveness, and prompted us to explore whether other mechanisms may operate.

### Loss of E-cadherin leads to the loss of global and nuclear β-catenin, but does not abolish Wnt responsiveness

If N-cadherin does not promote differentiation entirely through changes in cell cohesiveness, could it also be modulating pro-neural or anti-neural signalling pathways? This seems plausible because cadherins can bind various cell-surface signalling receptors and modulate signalling in other contexts (Bedzhov et al., 2012; Fedor-Chaiken et al., 2003; Greber et al., 2010).

We used a reverse phase protein array (RPPA) to measure the activity of a panel of signalling pathways (Table S2) and asked which of them are changed in response to cadherin switching. We assayed signalling pathway activity by measuring changes in the abundance of total protein levels and various phospho-protein species in Ecad^−/−^ cells and Ecad^Ncad/Ncad^ cells compared with their respective control cell lines. All cell lines were cultured in neural differentiation conditions for 24 h, a time prior to the emergence of the earliest neural cells that appear in response to either endogenous or experimentally induced cadherin switching.

**Fig. 4.**
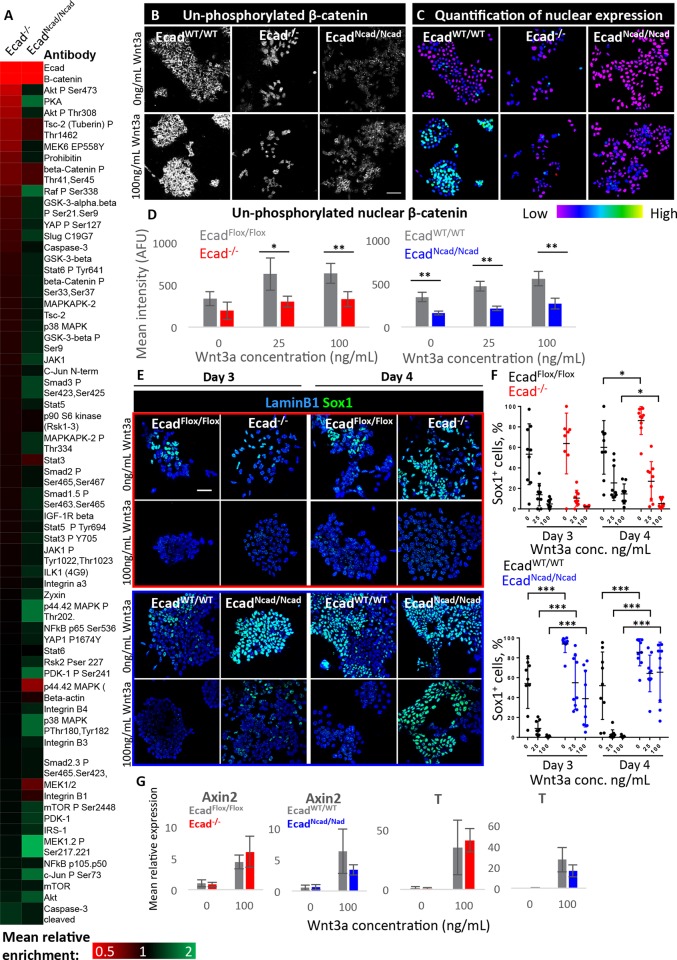
**Effects of cadherin switching on β-catenin and Wnt signalling.** (A) Heat map showing enrichment of protein and phospho-protein species in Ecad^−/−^ and Ecad^Ncad/Ncad^ cells compared with control cell lines; all cells were cultured in neural differentiation conditions for 24 h at time of analysis. Data generated by RPPA, *n*=3. (B) Example confocal images of cells at 24 h of neural differentiation cultured in varying Wnt3a concentration and stained for unphosphorylated β-catenin. (C) Quantitative visualisations of nuclear staining of the images shown in B. (D) Quantification of mean nuclear voxel intensity for unphosphorylated β-catenin in cells cultured for 24 h in neural differentiation conditions. *n*=4 biological replicates, each containing hundreds of cells. (E,F) Example images (E) of Sox1 expression in cells cultured with or without Wnt3a. ‘High Wnt3a’ refers to 100 ng/ml. Dot plots (F) show quantification of the percentage of Sox1-positive cells; each dot represents one field of view; *n*=9, three images each sampled from three biological replicates. (G) qPCR analysis of two Wnt pathway readouts in Ecad^−/−^ and Ecad^Ncad/Ncad^ cells during neural differentiation in increasing concentrations of Wnt3a. *n*=3 biological replicates. For all graphs, error bars represent s.d., *P≤0.05, **P≤0.01, ***P≤0.001; unpaired Student's *t*-test. Scale bars: 50 µm.

We first confirmed that the most significant change in protein abundance in Ecad^−/−^ and Ecad^Ncad/Ncad^ cells was a loss of E-cadherin, as expected. The next most significant change was a depletion of β-catenin, levels of which were reduced by 3-fold both in Ecad^−/−^ and Ecad^Ncad/Ncad^ cells compared with control cells (Fig. 4A). This is in keeping with reports that β-catenin expression is reduced in response to a reduction in E-cadherin in other contexts (Hendriksen et al., 2008; Van De Wetering et al., 2001). Ecad^−/−^ cells also have moderately reduced levels of PKA, AktP-Ser473 and AktP-Thr308, but in all cases levels of these proteins were restored to at least normal levels in Ecad^Ncad/Ncad^ cells, making it unlikely that the pro-neural phenotype could be attributed to these changes in this context. We therefore focused on β-catenin as a candidate for mediating the pro-neural effects of cadherin switching.

As β-catenin is a central player in the canonical Wnt signalling pathway (Clevers, 2006), and Wnt is an anti-neural signal (Aubert et al., 2002; Haegele et al., 2003), we assessed whether the reduced levels of β-catenin caused changes in Wnt signalling responsiveness during differentiation. We first measured nuclear accumulation of an unphosphorylated (transcriptionally active) form of β-catenin in response to Wnt3a (Clevers, 2006). Ecad^−/−^, Ecad^Ncad/Ncad^ and control cells were cultured for 24 h in serum-free media with varying concentrations of Wnt3a, and the amount of unphosphorylated β-catenin staining in the nucleus was then quantified. As expected, nuclear β-catenin increased in control cells in response to increasing concentrations of Wnt3a (Fig. 4B-D). Strikingly, Ecad^−/−^ and Ecad^Ncad/Ncad^ cells accumulated significantly less nuclear β-catenin compared with WT cells even at the highest dose of Wnt3a. These results show that Ecad^−/−^ cells display a dampened response to Wnt3a (at least at the level of β-catenin accumulation), and that this is not rescued by N-cadherin.

We next asked whether cadherin switching allowed cells to resist the anti-neural effects of Wnt signalling. We first confirmed that the addition of Wnt3a suppressed neural differentiation in both WT cells and Ecad^−/−^ cells, as previously reported (Aubert et al., 2002; Haegele et al., 2003) (Fig. 4E,F). In contrast, Ecad^Ncad/Ncad^ cells upregulated early neural markers even in the presence of high concentrations of Wnt3a, in keeping with the hypothesis that cadherin switching maintains neural potency by dampening Wnt signalling (Fig. 4E,F, Fig. S4).

However, to our surprise, the Wnt target genes Axin2 and T responded to Wnt3a in Ecad^Ncad/Ncad^ cells to a similar extent as control cell lines (Fig. 4G). This indicated that, despite differences in global and nuclear levels of β-catenin, cells lacking E-cadherin were able to activate Wnt target genes normally during neural differentiation, as has been reported to be the case in other contexts (Van De Wetering et al., 2001). We speculate that this could be explained if the reduced levels of nuclear β-catenin remain above the threshold required for an efficient transcriptional response.

It is particularly interesting that Ecad^Ncad/Ncad^ cells activated the neural marker Sox1 even in the presence of the anti-neural signal Wnt3a (Fig. 4E,F); this observation suggests that cadherins become particularly important for reinforcing differentiation in a suboptimal signalling environment. However, this pro-neural effect of cadherin switching did not seem to be explained by a dampening of Wnt responsiveness because the transcriptional response to Wnt remained intact.

### Cadherin switching dampens FGF signalling during neural differentiation

We next set out to determine which other signalling pathways are influenced by cadherin switching. Our RPPA assay indicated that a large number of proteins involved in signalling pathways were modulated in response to the concerted loss of E-cadherin and gain of N-cadherin (Fig. 4A). We previously established that exogenous N-cadherin reinforces neural differentiation (Figs 2D and 4F), and so in order to simplify our search we decided to focus on signalling pathways than are modulated by N-cadherin.

We used a Nanostring assay to focus on transcriptional readouts of a broad range of signalling pathways. We measured changes in signalling pathway activity 48 h after inducing N-cadherin expression during neural differentiation using our dox-inducible N-cadherin cell line (A2Lox-Ncad-HA) as this was the time point at which the pro-neural phenotype became clearly apparent.

Of 770 genes assayed (Table S4), only a small number were upregulated in response to N-cadherin. These include markers of early neuroepithelial cells (Hes5: upregulated 2.4-fold; Pax3: upregulated 2.3-fold; Jag1: upregulated 1.5-fold,) consistent with a pro-neural effect of N-cadherin (Table S4). In contrast, a much larger number of genes (129 out of 142 genes at 48 h post-induction) were significantly downregulated in response to N-cadherin overexpression. This suggests that N-cadherin generally suppresses rather than activates transcriptional responses to signalling pathways in this context.

**Fig. 5.**
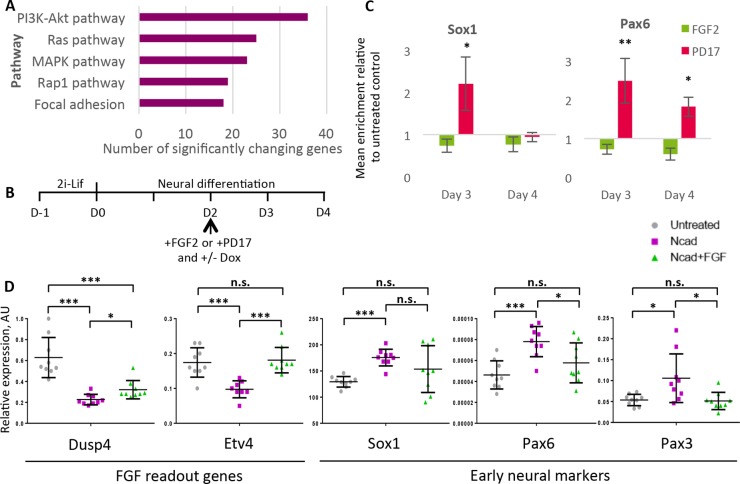
**Cadherin switching promotes neural differentiation by dampening FGF signalling.** (A) Top five signalling pathways most affected by N-cadherin overexpression 48 post-induction compared with un-induced control. Data generated by DAVID functional annotation of Nanostring mRNA expression values. 142 genes out of 770 genes tested changed significantly. (B) Protocol for FGF modulation experiments in inducible N-cadherin overexpressing cell lines. (C) qPCR analysis in WT cells with FGF2 or PD173074 (PD17). *n*=3 biological replicates, error bars represent s.e.m. *P≤0.05, **P≤0.01 compared with untreated control, unpaired Student's *t*-test. (D) qPCR analysis of gene expression after 48 h of N-cadherin induction. *n*=9, error bars represent s.d., *P≤0.05, ***P≤0.001, unpaired Student's *t*-test. n.s., no significance.

We then assigned each of these transcriptional changes to particular signalling pathways. This revealed that the top three pathways modulated by N-cadherin are PI3K/Akt, Ras and MAPK; all of these are pathways downstream of FGF receptors (Fig. 5A). These results indicated that N-cadherin dampens signalling pathways downstream of FGF during the early stages of neural differentiation, in keeping with reports that N-cadherin can interact with the FGF receptor in other contexts (Greber et al., 2010; Williams et al., 1994, 2001).

### N-cadherin promotes neural differentiation by dampening FGF signalling

We next assessed whether dampening of FGF signalling explains the pro-neural effect of cadherin switching.

It has previously been reported that FGF signalling promotes the acquisition and maintenance of primed pluripotency, but must then be downregulated in order for primed cells to progress to a neural fate (Greber et al., 2010; Jaeger et al., 2011; Stavridis et al., 2010; Sterneckert et al., 2010). In keeping with these reports, we found that blockade of the FGFR1 receptor using 100 ng/ml of the pharmacological inhibitor PD173074 enhances the efficiency of neural differentiation when added at D2. Conversely, addition of 20 ng/ml of FGF2 reduces expression of the early neural markers Sox1 and Pax6 when added at the same time point (Fig. 5B,C).

Having confirmed that FGF can act as an anti-neural signal in this context, we set out to test the hypothesis that N-cadherin promotes neural differentiation by dampening FGF responsiveness. In order to test this idea, we asked whether boosting FGF activity was able to reverse the pro-neural effect of N-cadherin.

We used dox-inducible N-cadherin (A2Lox-Ncad-HA) cells in order to induce N-cadherin overexpression during neural differentiation. FGF2 or the FGFR1 inhibitor PD173074 were added in order to modulate FGF activity. We added these reagents at D2, the same time that dox was added to induce N-cadherin (Fig. 5B). We used the FGF target genes Etv4 and Dusp4 to monitor FGF activity and demonstrated that N-cadherin dampens FGF activity during neural differentiation. We also observed that this effect could be at least partially rescued by addition of FGF2 (Fig. 5D). We then monitored neural differentiation by measuring expression of Sox1, Pax6 and Pax3. We found that addition of FGF2 reversed the pro-neural effects of N-cadherin, restoring the expression of these genes to similar levels as those seen in control cells that were undergoing differentiation in the absence of exogenous FGF or N-cadherin (Fig. 5D).

We conclude that N-cadherin can promote neural differentiation by dampening FGF signalling.

### Cadherin switching and neural differentiation are more synchronous *in vivo* than *in vitro*

N-cadherin is first detectable in a subset of undifferentiated epiblast stem cells prior to neural differentiation (Fig. 1). Cadherin switching then proceeds progressively over several days and is not completed in all cells until after neural fate is established. We next asked whether cadherin switching also occurs asynchronously over several days during neural development *in vivo*.

**Fig. 6.**
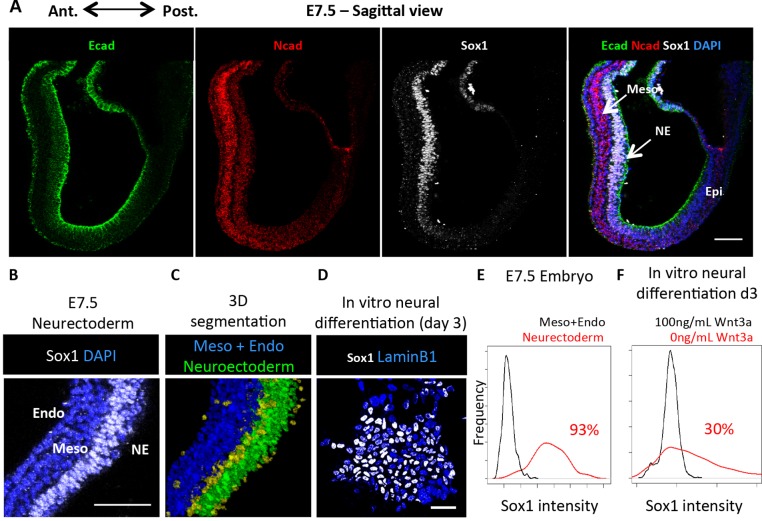
**Cadherin switching and neural differentiation are more synchronous *in vivo* than *in vitro*.** (A) E- and N-cadherin co-expression in an embryonic day (E)7.5 mouse embryo, sagittal view. (B) A region of Sox1^+^ neuroectoderm adjacent to Sox-negative mesoderm and endoderm is shown from an E7.5 embryo. Nuclei stained with DAPI. (C) Nuclear segmentation and binning of cells shown in B. Cells are assigned as belonging to the neuroectoderm layer (green) or non-neurectoderm layers (blue). Unassigned cells are shown in yellow. (D) Sox1 expression at day 3 of neural differentiation *in vitro*. Nuclei are stained with the nuclear envelope marker lamin B1. (E) Quantitative analysis of Sox1 expression in neural and non-neural tissues of the anterior E7.5 embryo. *n*=438 nuclei per sample. (F) Quantitative analysis of Sox1 expression at day 3 of neural differentiation *in vitro*, cultured with and without the neural differentiation inhibitor Wnt3a. *n*=231 nuclei per sample. In E and F, percentages refer to the proportion of Sox1-positive cells in the neural sample (red line; negative control population in black) as calculated by the non-overlapping area under the two curves. Endo, endoderm; Epi, epiblast; Meso, mesoderm; NE, neuroectoderm. Scale bars: 50 µm.

E-cadherin is expressed throughout the anterior epiblast during gastrulation. Although we were able to detect N-cadherin protein in EpiSC, we were unable to detect this adhesion molecule in the anterior epiblast of mouse embryos during gastrulation. N-cadherin protein is, however, readily detectable in mesoderm that has migrated from the posterior to lie adjacent to the anterior epiblast, as previously reported (Radice et al., 1997) (Fig. 6A, Fig. S5).

We then focused on the newly formed Sox1^+^ cells within the neural plate. We observed that this region uniformly displays E-cadherin but lacks detectable N-cadherin until after gastrulation (Fig. 6A, Fig. S5). In contrast to the heterogeneity in cadherin expression observed before and during neural differentiation in culture (Fig. 1A,H), we were unable to detect any obvious local cell-to-cell variability in expression of either E- or N-cadherin within the epiblast or the early neuroepithelium (Fig. 6A). We also noticed that almost every cell expresses Sox1 in the anterior neuroepithelium *in vivo*, whereas in contrast only around a third of cells express Sox1 during an equivalent stage of neural differentiation in culture (Fig. 6B-F).

We conclude that in the embryo, cells acquire N-cadherin after the acquisition of neural identity, consistent with a role in stabilising rather than inducing neural fate. We also observe that cadherin switching occurs relatively synchronously *in vivo*, in contrast with the asynchronous acquisition of N-cadherin in culture. Given our finding that N-cadherin can stabilise neural identity, this dysregulation of cadherin switching *in vitro* may help to explain why neural differentiation proceeds less synchronously in culture than in the embryo.

## DISCUSSION

Here, we report that the switch from E- to N-cadherin helps to reinforce neural commitment by dampening FGF signalling. It has previously been reported that premature cadherin switching results in gross morphological and cell-fate allocation defects at gastrulation, resulting at least in part from defects in extra-embryonic tissues (Basilicata et al., 2016). Our findings suggest that there may also be a cell-autonomous requirement for cadherin switching during neural differentiation.

E-cadherin is required to initiate differentiation in some contexts (Pieters et al., 2016), but once differentiation is triggered cadherins can have positive or negative effects on subsequent lineage specification (Pieters et al., 2016; Takehara et al., 2015), highlighting the multiple stage-specific effects of cadherins during differentiation of pluripotent cells. Our experiments focus on neural differentiation and so our data do not exclude the possibility that N-cadherin modulates differentiation into other lineages.

Our findings confirm previous reports that the absence of E-cadherin can limit the pool of nuclear β-catenin (Hendriksen et al., 2008; Orsulic et al., 1999; Van De Wetering et al., 2001), but we find that this does not result in a dampening of the transcriptional response to Wnt in differentiating neural progenitors; this is in keeping with similar findings in some cell types (Hendriksen et al., 2008; Orsulic et al., 1999; Van De Wetering et al., 2001), but contrasts with findings in other contexts where changes in E-cadherin do modulate the transcriptional response to Wnt (Howard et al., 2011; Libusova et al., 2010). Nevertheless, cadherin switching enables cells to resist the anti-neural effects of Wnt, possibly through an indirect mechanism. It would be interesting to explore the positional identity and potency of the Sox1^+^ cells that emerge in the presence of exogenous Wnt and N-cadherin, given that Sox1 is expressed in neuromesodermal progenitors (Cambray and Wilson, 2007) and that Wnt helps support neuromesodermal progenitor identity in the posterior of the embryo (Takemoto et al., 2005; Turner et al., 2014).

We find that N-cadherin can dampen FGF activity, leading us to speculate that N-cadherin might contribute to reinforcing neural commitment by protecting early neural cells from fluctuations in the anti-neural pro-mesoderm FGF signal. Because N-cadherin on one cell will stabilise N-cadherin on neighbouring cells through homotypic interaction, it is tempting to speculate that N-cadherin helps to propagate this neural-stabilisation effect through the tissue via a type of ‘community effect’. This could help ensure that neural commitment proceeds robustly in the embryo. It has recently been proposed that cadherins propagate mesodermal differentiation from cell to cell in an *in vitro* model of the primitive streak, although in that case communication is propagated predominantly through changes in E-cadherin rather than N-cadherin (Martyn et al., 2018). Differentiation is more variable and unpredictable in culture compared with the embryo, even though the extrinsic signalling environment in a culture dish can be tightly controlled. We speculate that a cadherin-mediated community effect may operate less efficiently in culture where the earliest N-cadherin-positive cells will often encounter neighbours that lack N-cadherin.

This work highlights the importance of changes in adhesion and morphology in ensuring robust development, and suggests that efforts to mimic these changes in culture will be crucial for gaining full control over differentiation of pluripotent cells.

## MATERIALS AND METHODS

### Animal care and use

Animal experiments (*Mus musculus* MF1) were performed under the UK Home Office project license PEEC9E359, approved by the Animal Welfare and Ethical Review Panel of the University of Edinburgh and within the conditions of the Animals (Scientific Procedures) Act 1986. The sex of embryos used in this study was not determined.

### Mouse ESC culture

#### Naïve stem cell culture (2i-Lif)

Naïve ESCs were maintained in 2i-Lif medium (Ying et al., 2008). This medium is N2B27 supplemented with 1 µM PD0325901, 3 µM Chiron 99021 and 100 units/ml LIF on laminin-coated tissue culture plates. Naïve stem cells were derived by passaging LIF-serum-cultured ESCs into 2i-Lif conditions, and maintaining them for at least three passages.

#### LIF-serum culture

ESCs were maintained in GMEM supplemented with 2-mercaptoethanol, non-essential amino acids, glutamine, pyruvate, 10% FCS and 100 units/ml LIF on gelatinised tissue culture flasks (Smith, 1991).

#### Epiblast stem cell culture

EpiSCs were maintained under published conditions (Brons et al., 2007; Tesar et al., 2007). Briefly, the cells were maintained in N2B27 solution supplemented with 20 ng/ml activin and 10 ng/ml FGF on fibronectin-coated tissue culture plates. EpiSCs were derived by passaging LIF-serum-cultured ESCs into EpiSC conditions, and maintaining them for at least three passages.

### Cell lines

E14tg2α mouse ESCs were used as WT cells. Previously published cell lines were generously provided by the researchers and labs who generated them: Sox1-GFP (‘46C’) cells (Aubert et al., 2003); Ecad^Ncad/Ncad^ cells (Basilicata et al., 2016; Kan et al., 2007), which were targeted to an E14.1 background, and chimeric mice were then backcrossed to C57BL/6 for at least three or four generations before homozygous Ecad^Ncad/Ncad^ ESCs were established from blastocysts; Ecad^−/−^ cells on an E14-IB10 background, in which exons 4 to 15 were Cre-excised *in vitro* (Derksen et al., 2006; Pieters et al., 2016).

To generate dox-inducible N-cadherin overexpressing ESCs, the inducible cassette exchange (ICE) method was used (Iacovino et al., 2011, 2014). Primers were designed to allow for the amplification of a DNA fragment containing the *Cdh2* gene C-terminally tagged with an influenza virus hemagglutinin (HA) tag; the whole construct was flanked by XhoI and NotI restriction sites. The construct was ligated into a pCR Blunt II Topo vector (Thermo Fisher Scientific). The *Cdh2-HA* insert was then ligated into a p2Lox-eGFP plasmid, replacing an *eGFP* sequence in this construct (Iacovino et al., 2009). The resulting p2Lox-Cdh2-HA plasmid was then nucleofected into A2LoxCre cells. The cells were then cultured for 10 days under G418 selection, and surviving clonal colonies were then expanded. Clones were then screened for Ncad and HA expression by immunocytochemistry (ICC), and three clones with high transgene expression were selected for use in further experiments.

All cell lines used in this study were tested to confirm the absence of mycoplama contamination.

### Differentiation protocols

Monolayer neural differentiation was performed by passaging 2i-Lif-cultured ESCs at low density into laminin-coated tissue culture plates. The cells were maintained in 2i-Lif for 24 h to allow the cells to properly adhere to the matrix. After 24 h, media were changed to N2B27 medium in which commercial N2 was replaced with 0.5% modified N2 (made in-house as described by Pollard et al., 2006). Media were changed every 1-2 days.

### qRT-PCR

Primers used for qRT-PCR are described in Table S1. All expression values were normalised to the geometric mean expression value of at least two of three housekeeping genes: *Tbp*, *Sdha* and *Ywhaz*.

### RPPA analysis

RPPA analysis was performed on nitrocellulose-coated slides as previously described (Macleod et al., 2017). Briefly, cells were washed with PBS and lysed in 1% Triton X-100, 50 mM HEPES (pH 7.4), 150 mM sodium chloride, 1.5 mM magnesium chloride, 1 mM EGTA, 100 mM sodium fluoride, 10 mM sodium pyrophosphate, 1 mM sodium vanadate, 10% glycerol, supplemented with cOmplete ULTRA protease inhibitor and PhosSTOP phosphatase inhibitor cocktails (Roche). Cleared lysates were serially diluted to produce a four-step doubling dilution series of each sample, which were spotted in technical triplicate onto nitrocellulose-coated slides (Grace Bio-Labs) under conditions of constant 70% humidity using an Aushon 2470 array platform (Aushon Biosystems). After hydration, slides were blocked using SuperBlock (TBS) blocking buffer (Thermo Fisher Scientific) and incubated with validated primary antibodies (all diluted 1:250 in SuperBlock; Table S2). Bound antibodies were detected by incubation with anti-rabbit DyLight 800-conjugated secondary antibody (New England BioLabs). Slides were analysed using an InnoScan 710-IR scanner (Innopsys), and images were acquired at the highest gain without saturation of the fluorescence signal. The relative fluorescence intensity of each array feature was quantified using Mapix software (Innopsys).

Primary antibodies used in this assay are listed in Table S2. In the case of the Sox1 antibody, we determined that this antibody does not cross-react with Sox2 based on a lack of signal in undifferentiated ESCs but we cannot exclude the possibility of cross-reactivity with other Sox family members. The linear fit of the dilution series of each sample was determined for each primary antibody, from which median relative fluorescence intensities (RFI) values were calculated to provide relative quantification of total protein and phosphoprotein abundance across the sample set. Finally, signal intensities were normalised to total protein loading for each sample by using readout from a Fast Green (total protein)-stained array slide. Enrichment values for EcKO and NcKI cells were normalised to those in relevant control cell lines, and a mean enrichment was then calculated for three biological replicates.

### Immunofluorescence and FACS

For immunofluorescence analysis, cells cultured on glass coverslips were fixed in 4% formaldehyde and incubated for at least 30 min in blocking buffer (PBS, 3% donkey serum and 0.1% Triton X-100). Primary antibodies were diluted in blocking buffer and applied for 1 h at room temperature or overnight at 4°C. After three washes in PBS, secondary antibodies conjugated to Alexa fluorophores (Life Technologies) were diluted at 1:1000 in blocking buffer and applied for 1 h at room temperature. The cells were washed at least three times and the coverslips were mounted with Prolong Gold Antifade Reagent (Life Technologies) on glass slides for viewing.

For antibody staining of live cells for flow cytometry, cells were incubated with relevant antibodies on ice in the dark for at least 15 min. Antibodies were diluted in FACS buffer (5% FCS in PBS). Flow cytometric analysis was performed using a BD Accuri flow cytometer. FACS was carried out on a FACS Aria cell sorter.

Embryos were fixed with 4% formaldehyde/PBS/0.1% Triton X-100 (Sigma-Aldrich) for 30 min, and quenched with 50 mM ammonium chloride. Cellular permeabilisation was carried out for 10 min in PBS/0.1% Triton X-100. The embryos were incubated in primary antibody in 3% donkey serum/PBS/0.1% Triton X-100 overnight, and subjected to three washes in PBS/0.1% Triton X-100. Secondary antibodies were applied subsequently for 2 h to overnight, followed by three washes in PBS/0.1% Triton X-100. Antibody information is given in Table S3. Embryos were then stained with DAPI (Biotium), mounted in PBS droplets covered with mineral oil in ‘microscope rings’, and imaged on a Leica SP8 confocal microscope. Alternatively, following staining, chimaeric embryos requiring quantification of immunostaining were dehydrated in methanol series in PBS/0.1% Triton X-100, clarified in 50% methanol/50% BABB (benzyl alcohol:benzyl benzoate 1:2 ratio, Alfa Aesar and Sigma-Aldrich), transferred into 100% BABB in glass capillaries and imaged on a Leica SP8 confocal microscope.

### Transcript enrichment analysis

Gene enrichment datasets generated by Nanostring were analysed using the associated NSolver 4.0 software. Functional annotation of gene lists was performed using the DAVID functional annotation online tool (Huang et al., 2008, 2009). A list of significantly changing genes was used as an input list, and all 770 genes tested in the Nanostring analysis (Table S4) were used as a background list.

### Quantitative image analysis

#### Quantification of membrane-bound protein staining

Where fluorescent signal was to be quantified at the single cell level, cells were imaged in 3D *z*-stacks on a Leica SP8 three-detector confocal microscope. For the quantification of membrane staining, cells were counted manually using Fiji/ImageJ software.

#### Quantification of nuclear protein staining

For the quantification of nuclear staining, PickCells software was used. Cells were segmented by nuclear content or nuclear membrane staining (using DAPI or the nuclear envelope marker lamin B1 staining, respectively) using the software's inbuilt NESSys nuclear segmentation module (Blin et al., 2019). Protein expression was then quantified as the mean pixel intensity in any given nucleus. Where staining quality did not allow for accurate nuclear segmentation, cells were manually designated as either positive or negative based on a single empirical threshold for all images generated from a single biological replicate.

#### Quantification of inter-nuclear edge distances

Inter-nuclear edge distance was calculated using PickCells software in cells segmented using nuclear membrane staining. The nearest neighbours for each nucleus were determined using Delaunay triangulation, and the distance between the membranes of nearest neighbours was calculated for each nucleus up to a distance of 40 μm.

### Statistical analysis

All experiments that were statistically analysed were performed with at least three independent biological replicates. Statistical significance was calculated using a paired or unpaired Student's *t*-test as appropriate.

## Supplementary Material

Supplementary information
