# Supplementary material for: N-cadherin stabilises neural identity by dampening anti-neural signals
Source: Development. 2019 Nov 8;146(21):dev183269. doi: 10.1242/dev.183269 (PMC6857587; doi:10.1242/dev.183269)
Supplement: Supplementary information [file develop-146-183269-s1.pdf]

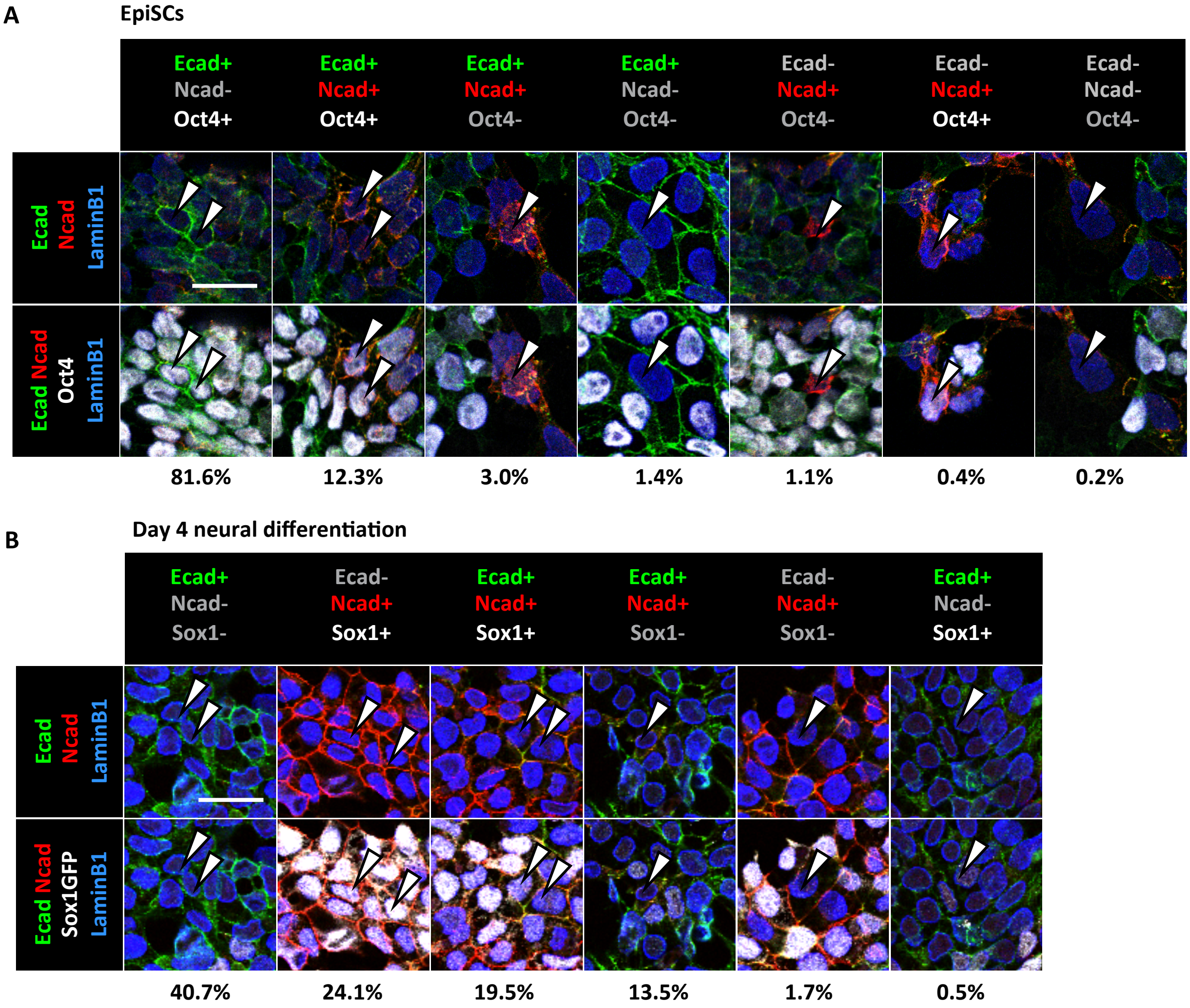

Figure S1: Single-cell views of protein co-expression in EpiSCs and during neural differentiation. White arrowheads point out individual cells of the given protein expression profile. Percentages below images indicate the proportion of cells of a given identity out of all cells analysed (manual quantification). Scale bars=25µm. **A.** EpiSCs stained for E-cadherin (green), N-cadherin (red), the pluripotency marker Oct4 (white) and nuclear envelope marker LaminB1 (blue). N=2596 ce lls from three biological replicates **B.** Cells on day four of neural differentiation from a 2i-Lif s tarting population stained for E-cadherin (green), N-cadherin (red), Sox1-GFP (shown in white), and nuclear envelope marker LaminB1 (blue). N=2275 cells from three biological replicates.

Development • Supplementary information

A: **Ecadh$^{Ncadh/Ncadh}$ cells**

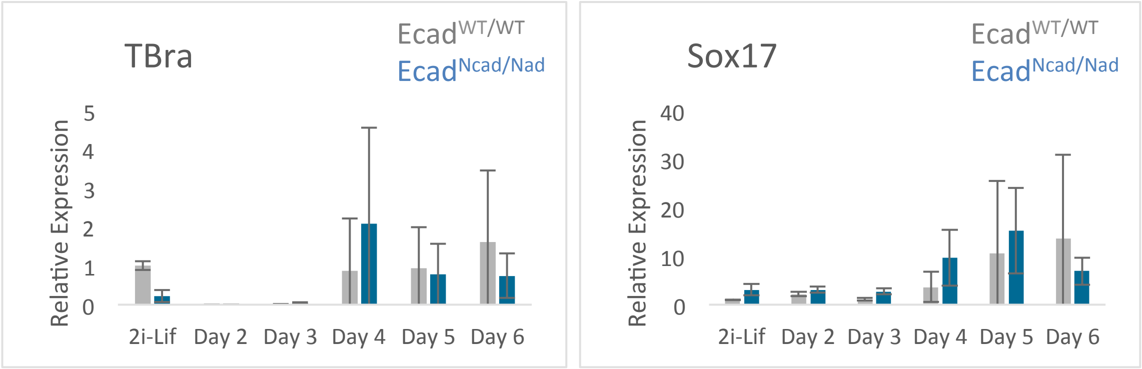

B: Dox-inducible N-Cadherin

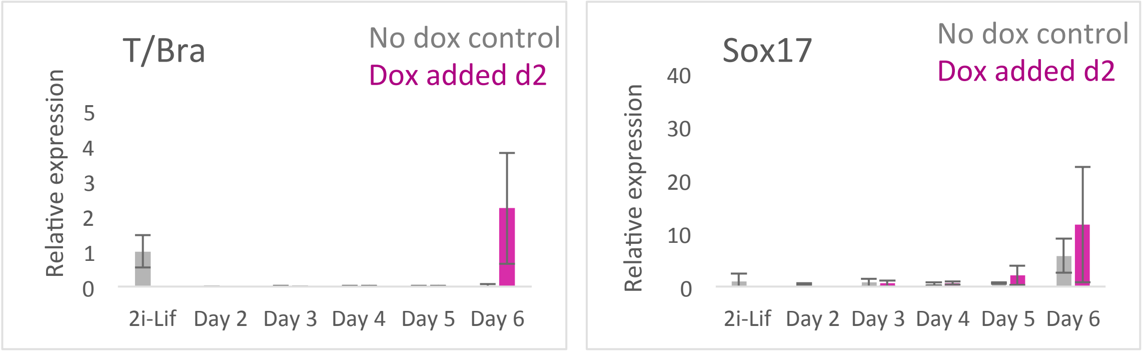

Figure S2: Cadherin switching has no clear effect on mesoderm or endoderm markers in the context of neural differentiation

A: qPCR analysis of Ecadh$^{Ncadh/Ncadh}$ cells during successive days of neural differentiation. N=3. Values normalised to control cell line on D0.

B: qPCR analysis of inducible N-cadherin overexpressing cells during neural differentiation; Dox added on day 2. N=9 (three biological replicates of three independent clones). Values normalised to control cell line on D0

Error bars=SD.

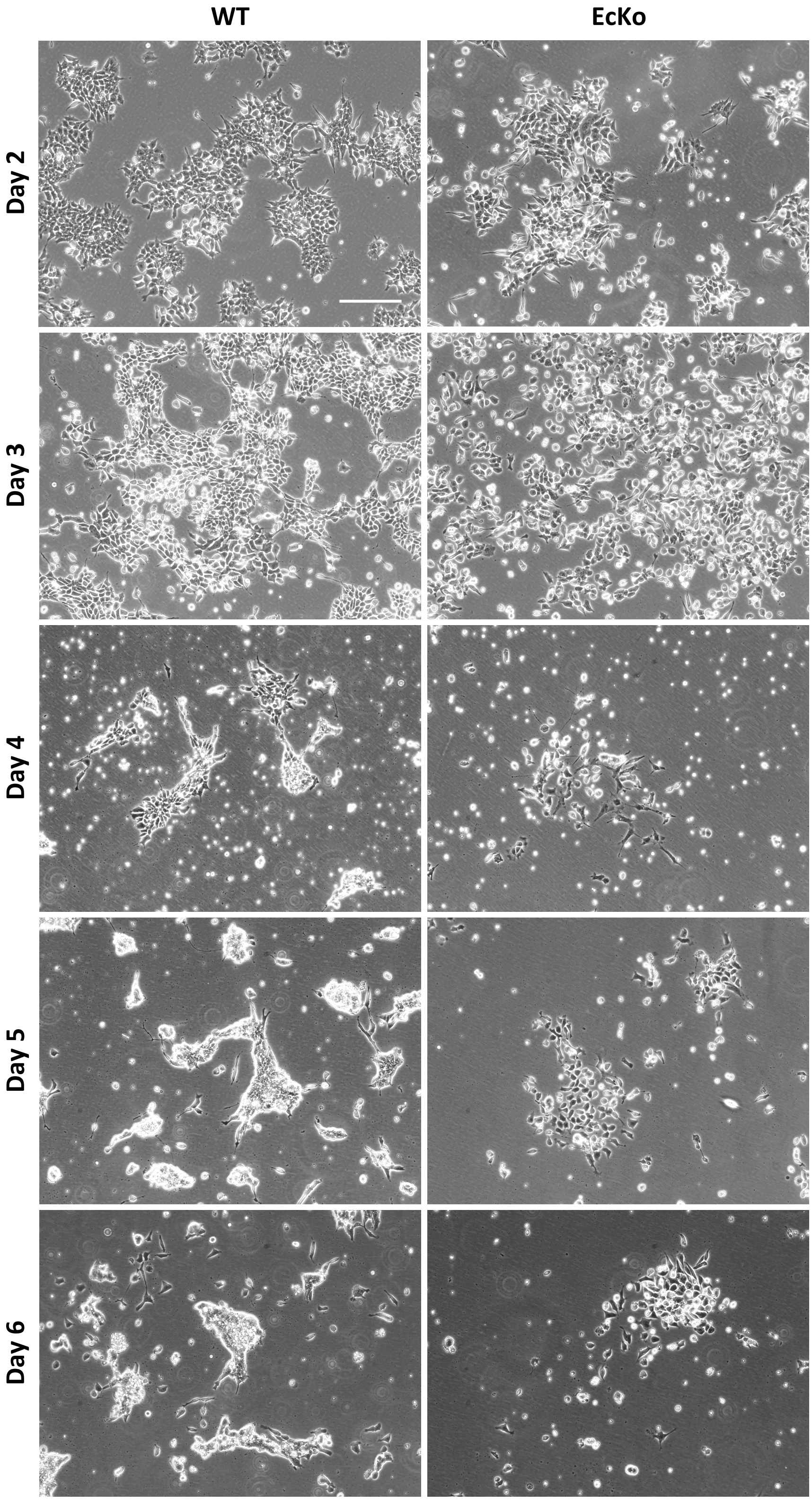

Figure S3: Ecad$^{-/-}$ cells die after prolonged culture in N2B27. Phase contrast images of Ecad$^{Flox/Flox}$ and Ecad$^{-/-}$ cells on days 2-6 of neural differentiation from a 2i-Lif starting population. Scalebar= 100µm.

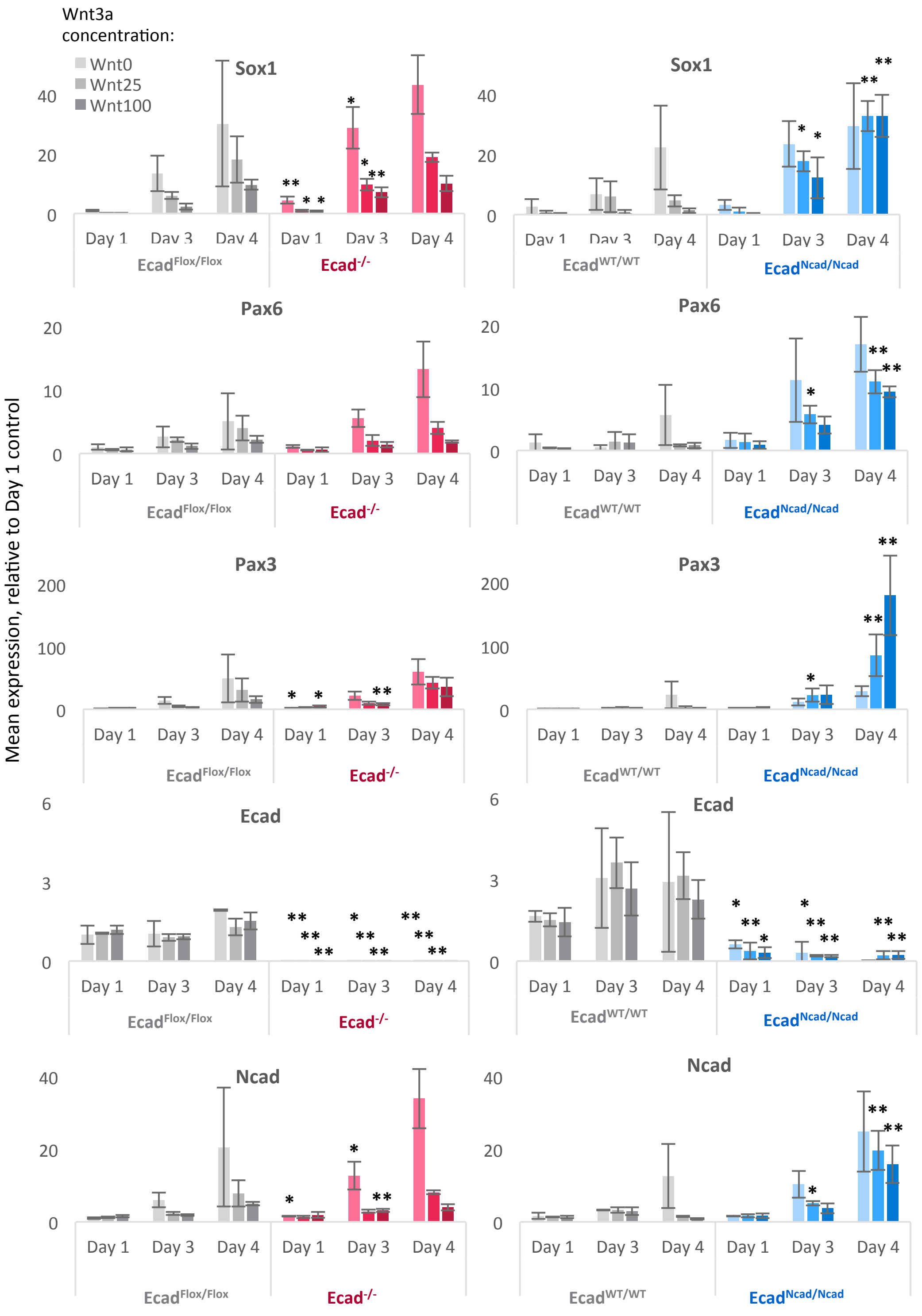

Figure S4: Effects of cadherin switching on β-catenin and WNT signalling. qPCR analysis of Ecad$^{Flox/Flox}$ and Ecad$^{Ncad/Ncad}$ cells during neural differentiation in increasing concentrations of Wnt3a; bars denote mean expression relative to the Day 1 condition of the relevant control cell line (grey). Asterisks denote significant difference compared to the paired control cell line in the same condition. N=3 biological replicates. Error bars=SD, *p≤0.05, **p≤0.01, paired T-test.

Development • Supplementary information

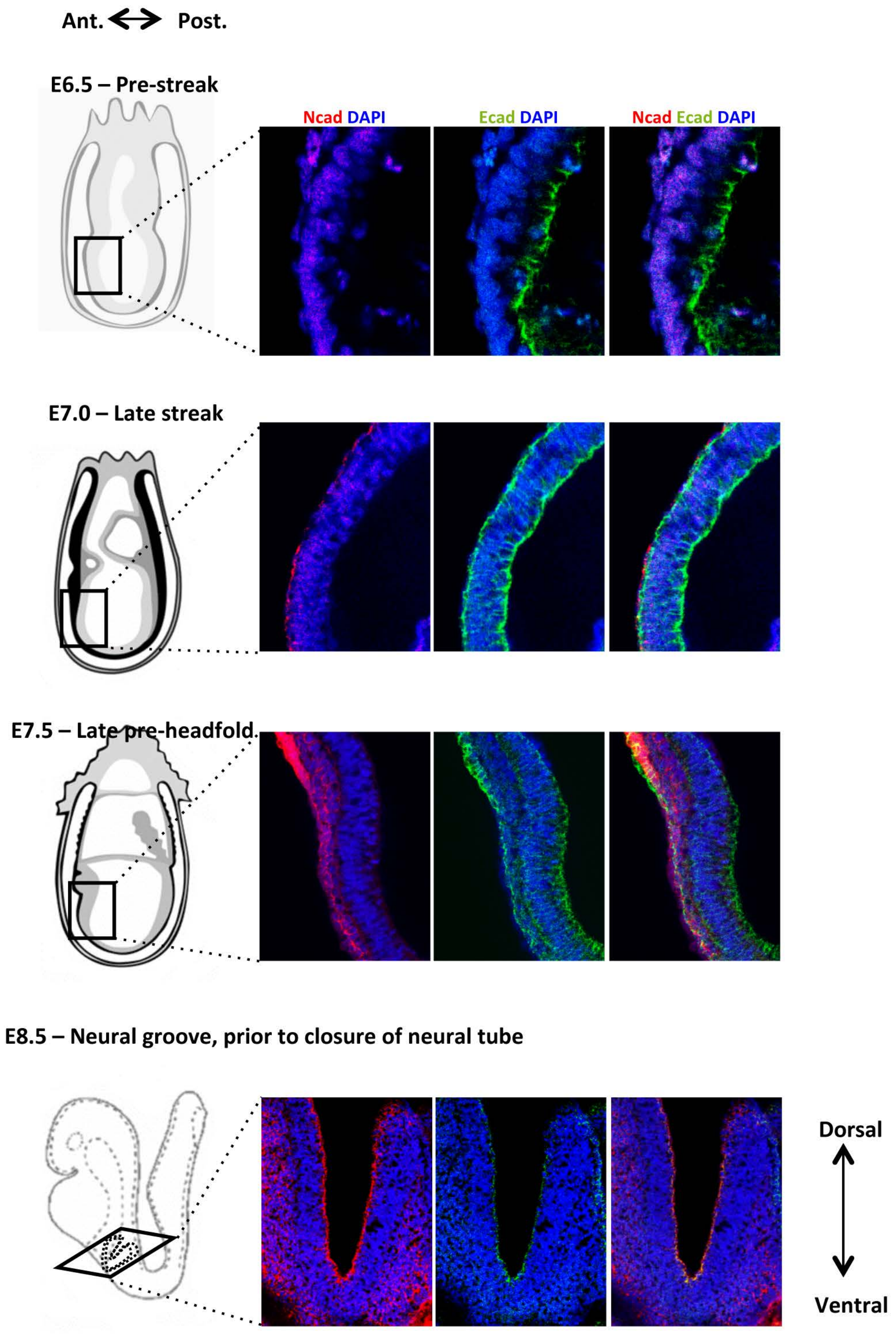

Figure S5: Cadherin switching in vivo. E-Cadherin (green) and N-Cadherin (red) immunostaining at various stages of mouse development as indicated. Embryo drawings are adapted from the EMAP eMouse Atlas Project (http://www.emouseatlas.org).

Development • Supplementary information

Table S1: qPCR primer sequences.

Primers used with the Universal Probe Library qPCR system from Roche.

Target gene	Forward sequence	Reverse sequence	UPL probe number
Ap2a	CCGGGTATTAACATCCCAGAT	CCGAAGAGGTTGTCCTTGTTA	94
Axin2	TGGGGAGCAGTTTTGTGC	CGGCTGACTCGTTCTCCT	96
Cdh1	ATCCTCGCCCTGCTGATT	ACCACCGTTCTCCTCCGTA	18
Cdh2	GCCATCATCGCTATCCTTCT	CCGTTTCATCCATACCACAAA	18
Dusp4	GCCTGGCCTACCTGATGAT	GCTGCTTGACGAACTCAAAA	25
Etv4	GGGTACCTTGGTGAGCACAG	CCCTGAGGAGATGTGAAGGA	66
Nanog	CCTCCAGCAGATGCAAGAA	GCTTGCACTTCATCCTTTGG	25
Pax3	AAAAGGCTAAACACAGCATCG	CAATATCGGAGCCTTCATCTG	110
Pax6	GTTCCCTGTCCTGTGGACTC	ACCGCCCTTGGTTAAAGTCT	78
Pou3f1	CTCAAGCCGCTGCTCAAC	CGCGATCTTGTCCAGGTT	25
Pou5f1	GTTGGAGAAGGTGGAACCAA	CTCCTTCTGCAGGGCTTTC	95
SDHA	CAGTTCCACCCCACAGGTA	TCTCCACGACACCCTTCTGT	71
Sox1	GTGACATCTGCCCCCATC	GAGGCCAGTCTGGTGTCAG	60
Sox17	CACAACGCAGAGCTAAGCAA	CGCTTCTCTGCCAAGGTC	97
T	ACTGGTCTAGCCTCGGAGTG	TTGCTCACAGACCAGAGACTG	27
TBP	GGGGAGCTGTGATGTGAAGT	CCAGGAAATAATTCTGGCTCA	97
Ywhaz	TTACTTGGCCGAGGTTGCT	TGCTGTGACTGGTCCACAAT	9

Development • Supplementary information

Table S2: RPPA antibodies

All antibodies were raised in rabbit

Epitope	Supplier	Catalogue no.
Akt	Cell Signaling Technologies	9272
Akt P Ser473	Cell Signaling Technologies	4060
Akt P Thr308	Cell Signaling Technologies	2965
beta-actin	Cell Signaling Technologies	4970
beta-Catenin	Cell Signaling Technologies	9562
beta-Catenin P Ser33,Ser37,Thr41	Cell Signaling Technologies	9561
beta-Catenin P Thr41,Ser45	Cell Signaling Technologies	9565
Caspase 3	Cell Signaling Technologies	9662
Caspase 3 cleaved	Cell Signaling Technologies	9664
c-Jun N-term	Epitomics	1254-1
c-Jun P Ser73	Cell Signaling Technologies	9164
E-Cadherin	Cell Signaling Technologies	3195
GSK-3-alpha/beta P Ser21/Ser9	Cell Signaling Technologies	9331
GSK-3-beta	Cell Signaling Technologies	9315
GSK-3-beta P Ser9	Cell Signaling Technologies	9336
IGF-1R beta	Cell Signaling Technologies	3027
ILK1 (4G9)	Cell Signaling Technologies	3856
Integrin alpha 4	Cell Signaling Technologies	4600
Integrin Beta 1 [EP1041Y]	Abcam	ab52971
Integrin beta3	Cell Signaling Technologies	4702
Integrin beta4	Cell Signaling Technologies	4707
IRS-1	Cell Signaling Technologies	2382
JAK1	Cell Signaling Technologies	3332
JAK1 P Tyr1022,Thr1023	Invitrogen (Biosource)	44-422G
MAPKAPK-2	Epitomics	1497-1
MAPKAPK-2 P Thr334	Cell Signaling Technologies	3041
MEK1/2	Cell Signaling Technologies	9122
MEK1/2 P Ser217/221	Cell Signaling Technologies	9154
MEK6 [EP558Y]	Abcam	ab52937
mTOR	Cell Signaling Technologies	2972
mTOR P Ser2448	Cell Signaling Technologies	2971
NFkB p105/p50	Calbiochem	GTX110585
NFkB p65 Ser536	Cell Signaling Technologies	3033
p38 MAPK	Cell Signaling Technologies	9212
p38 MAPK PThr180,Tyr182	Cell Signaling Technologies	9211
p44/42 MAPK (ERK1/2)	Cell Signaling Technologies	9102
p44/42 MAPK (ERK1/2) P Thr202/Thr185,Tyr204/Tyr187	Cell Signaling Technologies	4370
p90 S6 kinase (Rsk1-3)	Santa Cruz	sc-231
PDK-1	Cell Signaling Technologies	3062
PDK-1 P Ser241	Cell Signaling Technologies	3061
PKA	Abcam	ab26322
Prohibitin	Santa Cruz	sc-28259
Raf P Ser338	Cell Signaling Technologies	9427
Rsk2 Pser 227	Cell Signaling Technologies	3556
Slug (C19G7	Cell Signaling Technologies	9585
Smad1/5 P Ser463/Ser465	Cell Signaling Technologies	9516
Smad2 P Ser465,Ser467	Cell Signaling Technologies	3108
Smad2/3 P Ser465/Ser423,Ser467/Ser425	Cell Signaling Technologies	8828
Smad3 P Ser423,Ser425	Cell Signaling Technologies	9520
Stat3	Cell Signaling Technologies	12640
Stat3 P Y705	Cell Signaling Technologies	9131
Stat5	Invitrogen (Biosource)	44-368G
Stat5 P Tyr694	Cell Signaling Technologies	9351
Stat6	Cell Signaling Technologies	9362
Stat6 P Tyr641	Cell Signaling Technologies	9361
Tsc-2 (Tuberin)	Cell Signaling Technologies	3612
Tsc-2 (Tuberin) P Thr1462	Cell Signaling Technologies	3617
YAP P Ser127	Cell Signaling Technologies	4911
YAP1 [EP1674Y]	Abcam	ab52771
Zyxin	Cell Signaling Technologies	3553

Development • Supplementary information

Table S3: Immunocytochemistry and flow cytometry antibodies

All antibodies were diluted to the specified concentration in blocking buffer.

Epitope recognised	Clone	Host species	Dilution factor	Supplier	Cat. number
β-catenin (active), dephosphorylated on Ser37 or Thr41	8E7	Mouse	1:1000	Millipore	05-665
E-cadherin	DECMA-1	Rat	1:200	Sigma	U3254
E-cadherin, eFluor660-conjugated	DECMA-1	Rat	1:300	eBioscience	50-3249-82
GFP	Polyclonal	Chicken	1:1000	Abcam	13970
HA	HA-7	Mouse	1:1000	Sigma	H3663
Lamin β1	Polyclonal	Chicken	1:1000	Abcam	Ab90169
Lamin β1	Polyclonal	Rabbit	1:1000	Abcam	Ab16048
N-cadherin	32	Mouse	1:200	BD	610920
N-cadherin, AlexaFluor488-conjugated	Polyclonal	Sheep	1:50	R&D	FAB6426G
Oct4	N-19	Goat	1:200	Santa Cruz	SC-8628
Sox1	N23-844	Mouse	1:200	Pharmigen	560749

Table S4. Significant changes in gene expression 48 h after N-cadherin overexpression during neural differentiation Cells were cultured for 48 h in neural differentiation conditions when N-cadherin overexpression was induced by addition of Dox. RNA samples were collected for Nanostring gene expression analysis 48 h later. The analysis included 770 genes involves in cellular signalling pathways. Values show mean enrichment compared to un-induced controls. *N*=3 biological replicates.

Click here to Download Table S4
